# Suppression of Methylation-Mediated Transcriptional Gene Silencing by βC1-SAHH Protein Interaction during Geminivirus-Betasatellite Infection

**DOI:** 10.1371/journal.ppat.1002329

**Published:** 2011-10-20

**Authors:** Xiuling Yang, Yan Xie, Priya Raja, Sizhun Li, Jamie N. Wolf, Qingtang Shen, David M. Bisaro, Xueping Zhou

**Affiliations:** 1 State Key Laboratory of Rice Biology, Institute of Biotechnology, Zhejiang University, Hangzhou, People's Republic of China; 2 Department of Molecular Genetics, Plant Biotechnology Center, and Center for RNA Biology, The Ohio State University, Columbus, Ohio, United States of America; University of South Carolina, United States of America

## Abstract

DNA methylation is a fundamental epigenetic modification that regulates gene expression and represses endogenous transposons and invading DNA viruses. As a counter-defense, the geminiviruses encode proteins that inhibit methylation and transcriptional gene silencing (TGS). Some geminiviruses have acquired a betasatellite called DNA β. This study presents evidence that suppression of methylation-mediated TGS by the sole betasatellite-encoded protein, βC1, is crucial to the association of *Tomato yellow leaf curl China virus* (TYLCCNV) with its betasatellite (TYLCCNB). We show that TYLCCNB complements *Beet curly top virus* (BCTV) *L2^-^* mutants deficient for methylation inhibition and TGS suppression, and that cytosine methylation levels in BCTV and TYLCCNV genomes, as well as the host genome, are substantially reduced by TYLCCNB or βC1 expression. We also demonstrate that while TYLCCNB or βC1 expression can reverse TGS, TYLCCNV by itself is ineffective. Thus its AC2/AL2 protein, known to have suppression activity in other geminiviruses, is likely a natural mutant in this respect. A yeast two-hybrid screen of candidate proteins, followed by bimolecular fluorescence complementation analysis, revealed that βC1 interacts with S-adenosyl homocysteine hydrolase (SAHH), a methyl cycle enzyme required for TGS. We further demonstrate that βC1 protein inhibits SAHH activity *in vitro*. That βC1 and other geminivirus proteins target the methyl cycle suggests that limiting its product, S-adenosyl methionine, may be a common viral strategy for methylation interference. We propose that inhibition of methylation and TGS by βC1 stabilizes geminivirus/betasatellite complexes.

## Author Summary

Plants employ repressive viral genome methylation as an epigenetic defense against geminiviruses, and geminiviruses respond by elaborating proteins that inhibit methylation and transcriptional gene silencing (TGS). Some geminiviruses have acquired a satellite called DNA β (betasatellite), which depends on the helper virus for replication and spread within and between hosts. In return, the sole betasatellite encoded protein, βC1, encodes a pathogenicity factor that enhances viral replication and is responsible for inducing disease symptoms. Geminivirus/betasatellite complexes are common and cause significant losses of food and fiber crops. Here, we explore the molecular basis of the association between *Tomato yellow leaf curl China virus* (TYLCCNV) and its betasatellite (TYLCCNB). We show that TYLCCNV by itself is unable to reverse TGS. However, co-inoculation of TYLCCNB, or expression of βC1 protein, results in reduced methylation of both the helper virus and host genome, and reversal of TGS directed against a transgene and an endogenous locus. We also present evidence that βC1 accomplishes this by interacting with and inhibiting the activity of S-adenosyl homocysteine hydrolase (SAHH), an enzyme needed to maintain the methyl cycle that generates the methyltransferase co-factor S-adenosyl methionine. Thus, we propose that inhibition of methylation-mediated TGS by βC1 drives geminivirus/betasatellite association.

## Introduction

DNA methylation is a well-characterized epigenetic mark conserved in plants, animals, and some fungi that plays essential roles in a number of cellular processes, including the control of gene expression, the establishment of heterochromatin, paramutation, genomic imprinting, X-inactivation, transposon control, and transcriptional gene silencing (TGS) [1], [2], [3]. It also serves as a robust defense against geminiviruses [4], [5].

The *Geminiviridae* is a large family of viruses with circular, single-stranded DNA genomes that cause devastating diseases of economically important crops world-wide. The family includes four genera with the genus *Begomovirus* being the largest, containing more than 200 species [6], [7]. Many begomoviruses, such as *Cabbage leaf curl virus* (CaLCuV) and *Tomato golden mosaic virus* (TGMV), have two separately encapsidated genome components called DNA A and DNA B. However, some Old World begomoviruses, for example *Tomato yellow leaf curl China virus* (TYLCCNV), have a single genome component similar to DNA A of the bipartite viruses. In recent years, many monopartite begomoviruses have been found to acquire a betasatellite known as DNA β, a circular ssDNA about half the size (∼1.5 kb) of the helper virus genome. The betasatellite is typically responsible for the disease symptoms induced by the virus/satellite complex [8], [9], [10], [11]. Betasatellites in turn depend on the helper virus for replication, cell-to-cell and systemic spread through the host, and encapsidation and transmission to new host plants. DNA β encodes a single protein, βC1, that acts as a pathogenicity factor [12], [13]. To date, βC1 has been shown to suppress cytoplasmic post-transcriptional gene silencing (PTGS), a host defense that likely targets viral transcripts, and to interfere with host auxin and jasmonate signaling pathways [14], [15].

The small (2.5-3.0 kb) circular genomes of geminiviruses do not encode polymerases and instead depend on host systems for replication and transcription [16], [17]. Genomic ssDNA is replicated in the nucleus through double-stranded DNA intermediates that serve as replication and transcription templates and associate with cellular histones to form minichromosomes [18], [19]. These minichromosomes are potential targets for epigenetic repression, and recent studies have demonstrated that plants employ RNA-directed methylation leading to transcriptional gene silencing (TGS) as a defense against geminiviruses [20], [21]. As a counter-defensive measure, the AC2/AL2 proteins of the begomoviruses CaLCuV and TGMV, and the related C2/L2 proteins of *Beet curly top virus* (BCTV) and *Beet severe curly top virus* (BSCTV), both members of the genus *Curtovirus*, inhibit methylation and suppress TGS [22], [23]. Viral mutants lacking these proteins are unable to prevent hypermethylation of the viral genome, which suppresses virus replication and allows host plants to eventually recover from infection and produce asymptomatic shoots [20], [24]. One way that AC2/AL2 and C2/L2 suppress methylation is by inactivating adenosine kinase (ADK) [25], [26], [27]. ADK is required for maintenance of the methyl cycle that generates S-adenosyl methionine (SAM), a methyl group donor and essential methyltransferase co-factor [28]. In addition, the BSCTV C2/L2 protein has been shown to reduce the efficiency of SAM-dependent transmethylation by inhibiting proteasome-mediated degradation of S-adenosyl-methionine decarboxylase 1 (SAMDC1) [23]. SAMDC1 generates decarboxylated SAM (dcSAM), a competitive inhibitor of SAM.

In this study, we investigated whether the methylation pathway targets a monopartite begomovirus that associates with a betasatellite (TYLCCNV/TYLCCNB complex) and, if so, how the complex responds to this host defense. We show that TYLCCNV is subject to repressive cytosine methylation but, unlike most geminiviruses examined to date, it is unable to inhibit methylation-mediated TGS. Instead, the βC1 protein encoded by the betasatellite carries out this function. Furthermore, we present evidence that βC1 accomplishes this by interacting with S-adenosyl homocysteine hydrolase (SAHH), a methyl cycle enzyme required for SAM production and methylation-mediated TGS [29]. Using a novel *in vitro* assay, we demonstrate that βC1 inhibits SAHH activity.

## Results

### TYLCCNV methylation is reduced genome-wide by TYLCCNB

**Figure 1:**
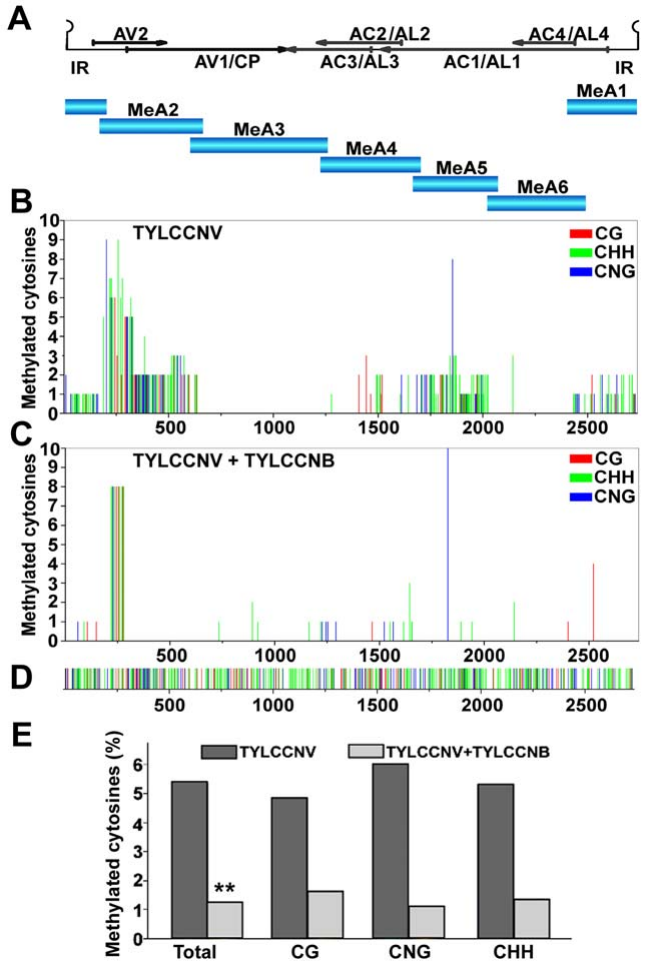
TYLCCNB reduces TYLCCNV DNA methylation genome-wide. *N. benthamiana* plants were inoculated with TYLCCNV alone or in combination with TYLCCNB. Samples were prepared by pooling six leaves from six systemically infected plants 21 days post-inoculation. DNA extracts were obtained, and the circular viral genome was divided into six fragments and PCR amplified after bisulfite treatment. Twenty clones from each fragment were sequenced. (A) The TYLCCNV genome is depicted in linear form, beginning within the conserved hairpin located in the intergenic region (IR). The positions of viral genes are shown by arrows. The six fragments used for bisulfite sequencing are indicated (MeA1 to MeA6). (B) The graph shows the total number of methylated cytosines at each position in the TYLCCNV genome, from a total of 20 sequenced clones, in different sequence contexts (CG red, CHH green, CNG blue). (C) As in (B), except DNA was obtained from plants co-inoculated with TYLCCNV + TYLCCNB. (D) The sequence contexts of all cytosines in the ∼2,737 bp TYLCCNV strain Y10 genome are indicated. (E) The histogram shows the percentage of cytosine residues methylated in different sequence contexts in the TYLCCNV genome in plants inoculated with TYLCCNV alone or together with TYLCCNB. Student's *t* test was performed on methylation values using individual clones as data points. The double asterisks indicate a significant difference between samples with and without TYLCCNB at the 99% confidence interval.

To determine whether TYLCCNV DNA is subject to methylation, bisulfite sequencing was carried out to analyze the methylation status of the entire viral genome. DNA extracts were obtained from TYLCCNV-infected *Nicotiana benthamiana* plants, and the genome was divided into six fragments using appropriate restriction endonucleases (Figure 1A). Following treatment with bisulfite reagent to convert unmethylated cytosines to uracil, the viral (plus) strand was amplified by PCR, cloned, and sequenced. Primers were targeted to sequences containing few cytosines, and were biased for templates with relatively low methylation density to examine the propensity for methylation at individual sites.

Sequencing of bisulfite-modified DNA revealed a mixture of unmethylated and methylated clones, with approximately a third of the clones methylated to some extent. The diversity of methylation patterns observed suggested that the 20 sequences obtained for each genome fragment originated from unique templates (Figure S1). Thus, similar to BCTV and CaLCuV, both methylated and unmethylated (or hypomethylated) viral genomes co-exist in TYLCCNV infected plants [20].

Interestingly, when cytosine methylation was mapped to the viral genome, relatively dense methylation at both CG and non-CG sites was mostly found at sequences encompassing or adjacent to viral promoters, as judged by transcript mapping and other data obtained with related geminiviruses. These include the intergenic region (IR) that contains divergent promoters flanking the origin of replication [30], [31], [32], the downstream complementary sense promoters within the AC1/AL1 coding region that drive expression of AC2/AL2 and AC3/AL3 [33], [34], and sequences within the AV2 and AV1 reading frames that include the AV1/CP (coat protein) promoter (Figure 1B). To our knowledge, this is the first genome-wide analysis of geminivirus genome methylation at single base resolution. The results provide direct evidence that TYLCCNV is targeted by cytosine methylation, and that methylation appears to be enriched at sequences within or near promoters.

To determine whether the betasatellite impacts helper virus methylation, TYLCCNV methylation status was also analyzed following co-inoculation of TYLCCNV and TYLCCNB. In the presence of the betasatellite, cytosine methylation of the TYLCCNV genome was rare and mostly limited to the AV2 region, with only occasional methylation at other locations (Figure 1C). This AV2 sequence was previously identified as a hotspot for siRNA production, although why it is a particularly attractive target for the silencing machinery is not clear [35]. Apart from this sequence, TYLCCNV DNA methylation was reduced in all contexts by TYLCCNB. Total methylation was only ∼1.25% of cytosines, considerably lower than the ∼5.4% detected in plants infected with TYLCCNV alone (Figure 1E). Student's *t* test confirmed that this difference is significant at the 99% confidence interval. We concluded that TYLCCNB substantially reduces genome-wide methylation of the TYLCCNV helper virus in infected plants.

### TYLCCNB complements BCTV *L2^-^* mutants and prevents host recovery

The goal of this study was to ascertain whether TYLCCNB βC1 has a function similar to BCTV L2 (C2) protein, which is known to inhibit methylation and whose absence permits hosts to recover from infection. Disease symptoms elicited in *N. benthamiana* or *Arabidopsis* plants by BCTV or a BCTV *L2^-^* null mutant are similar in primary infected tissue. However, after primary tissue is harvested, new secondary shoots continue to show severe symptoms in BCTV-infected plants, but exhibit recovery (symptom remission) in plants infected with BCTV *L2^-^* [36]. The small RNA-directed methylation pathway is required for host recovery from BCTV *L2^-^* infection, and the majority of viral genomes in recovered tissue are hypermethylated [20].

**Figure 2:**
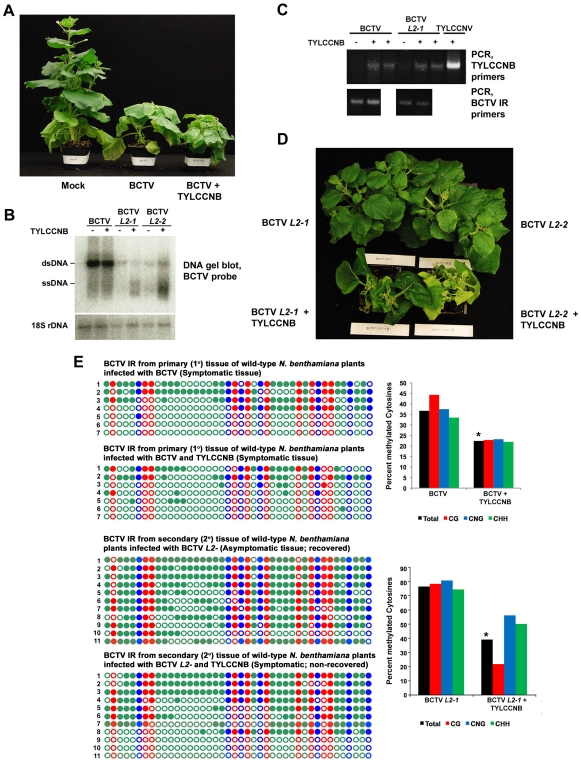
TYLCCNB complements BCTV *L2^-^* mutants. (A) TYLCCNB imparts characteristic symptoms when co-inoculated with BCTV. *N. benthamiana* plants were mock inoculated, or inoculated with BCTV or BCTV + TYLCCNB and photographed 21 to 30 days later. (B) DNA gel blot hybridization analysis of BCTV DNA from primary tissue, and BCTV *L2-1* and *L2-2* DNA from recovered or non-recovered secondary tissue. BCTV and BCTV *L2^-^* mutants were inoculated alone or with TYLCCNB, as indicated. DNA extracts were prepared from pools of at least six plants and digested with ScaI to linearize the viral genome and cleave host genomic 18S rDNA to provide a loading control. Viral single- and double-stranded DNAs (ssDNA, dsDNA) are indicated. (C) PCR detection of BCTV and TYLCCNB DNA in infected plants. An ethidium bromide stained gel of PCR products obtained with TYLCCNB- or BCTV intergenic region (IR)-specific primers is shown. Plants were inoculated as indicated. (D) TYLCCNB complements BCTV *L2^-^* mutants and prevents host recovery. Plants were inoculated with BCTV *L2-1* or *L2-2* mutants alone, or with TYLCCNB. Symptomatic, primary infected tissues were removed 21-30 days post-inoculation, after which secondary shoots were allowed to grow for 14-21 days. Plants were photographed from above to reveal the extent of recovery in virus inoculated plants, and absence of recovery in co-inoculated plants. (E) Cytosine methylation profiles confirm that TYLCCNB inhibits methylation. Plants were inoculated, and viral DNA was obtained from symptomatic primary tissue, and recovered or non-recovered secondary tissue, as indicated. Methylation of the BCTV IR was assessed by bisulfite sequencing. The circles represent cytosines in the IR and are color coded according to sequence context (CG red, CHH green, CNG blue). Filled circles indicate methylation, and each line represents the sequence of an individual clone. Graphic representations of the same data, with percent methylated cytosines on the Y-axis, are shown to the right. Student's *t* test was performed on total methylation values using individual clones as data points. Asterisks indicate significant differences between samples with and without TYLCCNB at > 95% confidence.

We first established that BCTV can support betasatellite replication by co-inoculating *N. benthamiana* plants with BCTV and TYLCCNB. BCTV causes severe stunting and upward leaf curl in this host. However, in co-inoculated plants, stunting was accompanied by a downward leaf roll characteristic of symptoms induced by TYLCCNB with its cognate helper virus (Figure 2A). TYLCCNB did not significantly enhance BCTV DNA levels, as determined by DNA gel blot analysis (Figure 2B), and it proved difficult to detect TYLCCNB by this method. However, PCR amplification with TYLCCNB-specific primers revealed that the betasatellite was present in systemically infected leaves of BCTV + TYLCCNB inoculated plants, albeit at low levels compared to TYLCCNV + TYLCCNB infections (Figure 2C). Nevertheless, these experiments show that TYLCCNB can be replicated by BCTV, and to our knowledge provide the first evidence of betasatellite replication by a curtovirus.

We next examined secondary infected tissues of plants inoculated with BCTV *L2^-^* or BCTV *L2^-^* + TYLCCNB. Two different null mutants (*L2-1* and *L2-2*) were used in these experiments [36]. As expected, the secondary shoots of plants inoculated with the *L2^-^* mutants recovered from infection (30 plants each treatment), and the asymptomatic tissue contained reduced amounts of viral DNA (Figure 2B and 2D). By contrast, plants co-inoculated with BCTV *L2-1* or *L2-2* + TYLCCNB did not recover and secondary shoots exhibited severe symptoms (30 plants each treatment) (Figure 2D). The non-recovered tissues contained moderately increased amounts of viral DNA, particularly viral ssDNA, relative to plants inoculated with mutant virus alone, and TYLCCNB could be detected by PCR (Figure 2B and 2C). Similar increases in viral DNA levels were observed previously in symptomatic secondary shoots of *ago4* plants inoculated with BCTV *L2^-^* mutants, which lack the ability to recover [20]. We concluded that TYLCCNB effectively complemented the BCTV *L2^-^* mutants, providing genetic evidence that the βC1 and L2 proteins have similar functions.

Cytosine methylation was directly examined by bisulfite sequencing of the ∼300 bp BCTV IR. Despite low abundance of the betasatellite, we observed that methylation in primary infected tissue was reduced by about 15% in BCTV + TYLCCNB compared to BCTV infected plants (Figure 2E). As seen in earlier studies, nearly all viral genomes from recovered secondary tissue of plants infected with BCTV *L2-1* were hypermethylated (total methylation ∼75%) [20]. However, methylation was reduced to about 40% in non-recovered, secondary shoots from BCTV *L2-1* + TYLCCNB infected plants. In both cases, the differences between samples with and without TYLCCNB were highly significant (p>95%). These results suggest that, like L2, the βC1 protein prevents host recovery by inhibiting methylation, providing further evidence that an important role of TYLCCNB is to reduce methylation of the helper virus genome.

### βC1 protein causes global reductions in host genome cytosine methylation

To assess the effect of TYLCCNB on host DNA, a previously described cytosine extension assay was used to examine the methylation status of the *N. benthamiana* genome following infection with TYLCCNV or TYLCCNV + TYLCCNB [22], [37]. Total DNA obtained from inoculated plants was digested with the methylation sensitive endonuclease MspI, whose cleavage activity is blocked by methylation of the external cytosine in its target site (C ^↓^CGG). Following digestion, a single-nucleotide extension assay was performed using ^32^P-dCTP and Taq DNA polymerase, which lacks a proofreading activity. Under these conditions, nucleotide incorporation reflects the number of CCGG sites cleaved by MspI, which in turn is negatively correlated with methylation at CNG sites within the genome.

**Figure 3:**
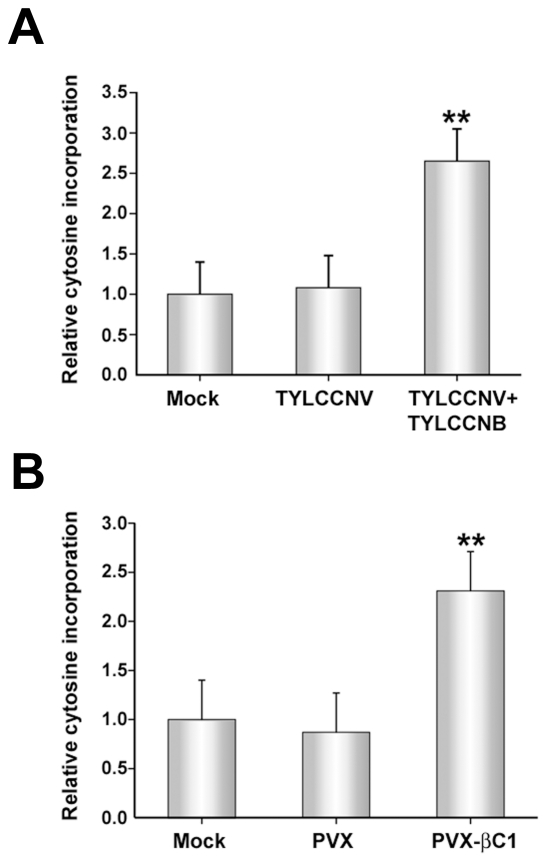
TYLCCNB, or βC1 expression, globally reduces cytosine methylation in the host genome. The histograms depict the relative ^32^P-dCTP incorporation observed in methylation-sensitive extension assays. DNA was obtained from mock inoculated or infected plants, digested with MspI, and incubated with ^32^P-dCTP and Taq polymerase to permit single-nucleotide extension. Increased incorporation due to enhanced MspI cleavage indicates reduced methylation. (A) DNA was obtained from mock-inoculated plants, or plants inoculated with TYLCCNV or TYLCCNV + TYLCCNB. (B) DNA was obtained from mock-inoculated plants, or plants inoculated with PVX or PVX expressing βC1 protein (PVX-βC1). Both (A) and (B) represent data from two independent experiments. In each experiment, assays were performed in duplicate with DNA from three individual plants per treatment. Values represent means +/- SE. Double asterisks indicate significant differences between samples with and without TYLCCNB at the 99% confidence interval, as determined by Student's *t* test.

Cytosine extension assays showed no apparent difference between CNG methylation levels in DNA from mock- and TYLCCNV-inoculated *N. benthamiana* plants. Remarkably, however, a 2.5-fold increase in the incorporation of labeled cytosine was observed with DNA from plants co-inoculated with TYLCCNV and TYLCCNB (Figure 3A). Student's *t* test confirmed that this difference was significant at the 99% confidence interval. Thus, the betasatellite enhanced MspI cleavage, revealing its ability to globally reduce methylation at the CNG sites queried in the context of a natural infection.

**Figure 4:**
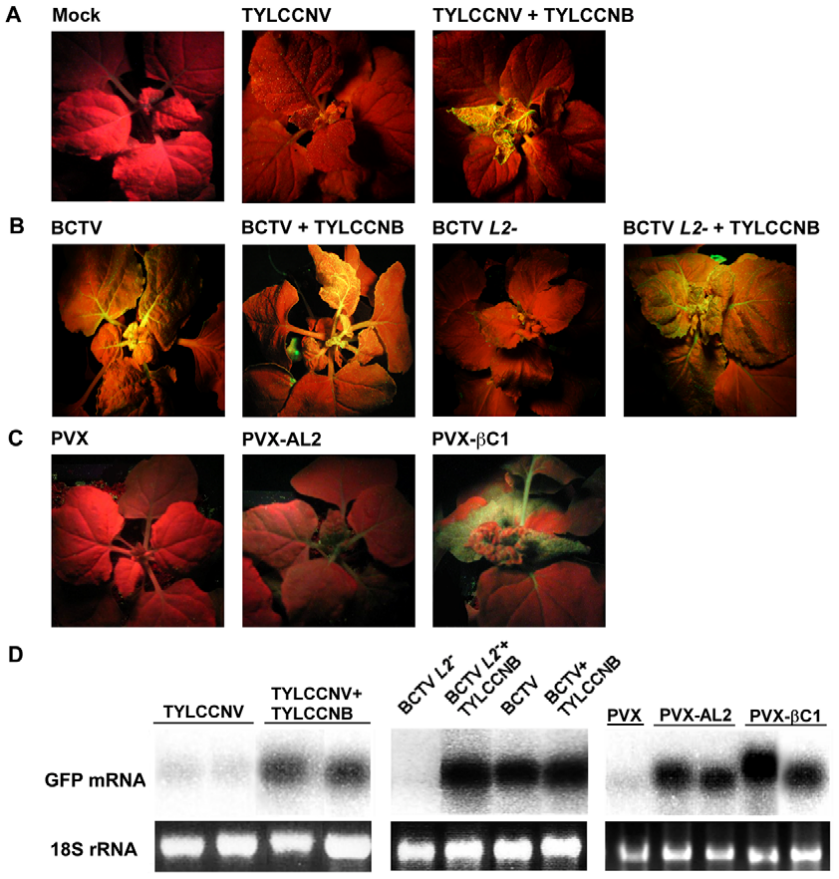
TYLCCNB, or βC1 expression, reverses TGS of a GFP transgene. In experiments with TYLCCNV and PVX, plants were photographed under UV light 14 days post-inoculation. In experiments with BCTV, plants were photographed after 21 days due to the longer latent period of this virus. Results shown are representative of at least three independent experiments with 4 to 8 plants per treatment. (A) *N. benthamiana* plants containing a transcriptionally silenced GFP transgene (line 16-TGS) were mock inoculated, inoculated with TYLCCNV, or co-inoculated with TYLCCNV and TYLCCNB. (B) Line 16-TGS plants were inoculated with BCTV, BCTV *L2-2 (L2^-^)*, or co-inoculated with these viruses and TYLCCNB. (C) Plants were inoculated with the PVX vector, PVX expressing AL2 from CaLCuV (PVX-AL2, positive control), or PVX expressing βC1 (PVX-βC1). GFP expression in the PVX-AL2 (CaLCuV) plant shown was less intense than is typically observed [22]. (D) Gel blot analysis of RNA from leaves of 16-TGS plants inoculated as indicated. The ^32^P-labeled probe was specific for GFP mRNA. The 18S rRNA loading controls were visualized by ethidium bromide staining.

Experiments described to this point have demonstrated that the presence of TYLCCNB results in reduced methylation levels in both helper virus and host genomes. Since βC1 is the only protein encoded by the betasatellite, we asked whether the methylation inhibition activity is attributable to this protein. To do this, a *Potato virus X* (PVX) expression vector was employed to express βC1 [38]. *N. benthamiana* plants were mock-inoculated or inoculated with PVX (empty vector, negative control) or PVX expressing βC1 (PVX-βC1). PVX infection induced typical mild mosaic symptoms in systemically infected leaves. However, by 14 days post-inoculation (dpi), plants infected with PVX-βC1 displayed dramatically intensified symptoms, including enations and downward leaf curl similar to that seen in TYLCCNV + TYLCCNB infected plants (see Figure 4). Cytosine extension assays showed no significant difference in CNG methylation in DNA isolated from mock- and PVX-inoculated plants. However, nearly a 2.5-fold increase in nucleotide incorporation was evident with DNA from PVX-βC1 inoculated plants, indicative of a similar reduction in host genome-wide CNG methylation (Figure 3B). We concluded that βC1 is responsible for the methylation reductions caused by TYLCCNB.

### βC1 protein reverses TGS of a GFP transgene

We next asked whether TYLCCNV, or its betasatellite, have the ability to suppress TGS. These experiments employed *N. benthamiana* line 16-TGS, which contains a transcriptionally silenced green fluorescent protein (GFP) transgene flanked by the *Cauliflower mosaic virus* 35S promoter. This assay was used previously to demonstrate that CaLCuV, TGMV, and BCTV, or their AC2/AL2 or C2/L2 proteins, can suppress TGS [5], [22]. Here 16-TGS plants were inoculated either with TYLCCNV alone, or with TYLCCNV and TYLCCNB. Under UV light, plants in which GFP is not expressed appear red due to chlorophyll autofluorescence, whereas regions expressing GFP appear yellow-green.

By 14 dpi, TYLCCNV infected plants showed very mild symptoms, while as expected severe symptoms were evident in plants infected with TYLCCNV + TYLCCNB. In addition, yellow-green GFP fluorescence was visible in symptomatic leaves of the co-inoculated 16-TGS plants. Surprisingly, no fluorescence was evident in plants infected with TYLCCNV alone (Figure 4A). RNA gel blot analysis verified that the visible fluorescence in TYLCCNV + TYLCCNB co-inoculated plants was accompanied by the accumulation of GFP mRNA (Figure 4D). These results demonstrate that TYLCCNB can reverse established TGS in the context of a natural infection, and that the TYLCCNV helper virus lacks suppression activity even though it encodes an AC2/AL2 protein. To rule out the possibility that lack of suppression by TYLCCNV was due to low levels of AC2/AL2 protein accumulation, this protein was expressed in 16-TGS plants using a PVX vector. We found that PVX-AC2 (TYLCCNV) was also unable to reverse TGS, indicating that TYLCCNV lacks significant TGS suppression activity (Figure S2).

That TYLCCNB can suppress TGS was confirmed by its ability to complement BCTV *L2-2*. As demonstrated previously, BCTV reversed TGS in an L2-dependent manner [22], and the combination of wild-type BCTV and TYLCCNB did not result in a substantial increase in TGS suppression over that seen with BCTV alone. However, TYLCCNB was able to complement the silencing-defective BCTV *L2^-^* mutant, as yellow-green GFP fluorescence and GFP mRNA were clearly evident in plants co-inoculated with BCTV *L2^-^* and TYLCCNB (Figure 4B and 4D).

We next confirmed that βC1 was responsible for the suppression activity seen with TYLCCNB by expressing the protein from a PVX vector. In this experiment, 16-TGS plants were inoculated with PVX (empty vector control), PVX-AL2 (CaLCuV AL2, positive control), or PVX-βC1. PXV infection had no effect on GFP silencing. By contrast, plants inoculated with PVX-βC1 displayed dramatically intensified PVX symptoms accompanied by bright yellow-green fluorescence (Figure 4C). The enhancement of symptoms and fluorescence seen with PVX-βC1 was even more dramatic than that elicited by the PVX-AL2 positive control. RNA gel blot analysis confirmed that TGS reversal was accompanied by the accumulation of GFP mRNA (Figure 4D). We concluded that βC1 protein is responsible for the TGS suppression activity observed with TYLCCNB.

### Transgenic expression of βC1 can reverse endogenous, epigenetic gene silencing

To confirm TGS suppression activity, transgenic *Arabidopsis* lines expressing βC1 from a dexamethasone (dex)-inducible promoter were constructed (dex-βC1). We previously used a similar approach to examine the suppression activities of the CaLCuV AC2/AL2 and BCTV C2/L2 proteins [22]. The dex-βC1 transgenes were verified by genomic PCR, and transgene expression was monitored by RNA gel blots. Multiple independent lines expressing high levels of βC1 following dex treatment were selected for further analysis. Extracts from three of these lines were used for semi-quantitative reverse transcriptase PCR (sqRT-PCR) with primers to amplify transcripts from a putative *F-box* locus (At2g17690) known to be silenced by methylation, or from a control gene (*Actin*) [39], [40].

**Figure 5:**
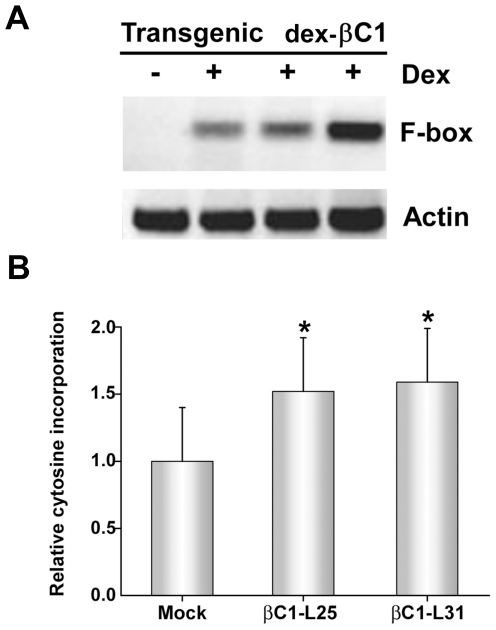
Transgenic expression of βC1 inhibits methylation and suppresses epigenetic TGS. (A) *Arabidopsis* plants from three independent lines containing a dex-inducible βC1 transgene (dex-βC1) were treated with dex (+) or mock-treated (-), and expression of a putative *F-box* locus known to be methylated and transcriptionally silenced was assessed by sqRT-PCR. Actin served as a control. Mock treated samples were prepared by pooling RNA from the three transgenic lines. Data shown are representative of at least three independent experiments. (B) The histogram illustrates relative incorporation observed in the methylation-sensitive extension assay using MspI. Extracts from two transgenic lines (βC1-L25 and βC1-L31) were tested following dex treatment. Two independent experiments were performed. In each experiment, assays were performed in duplicate with DNA from three individual plants per treatment. Mock treatment assays contained DNA isolated from plants of both transgenic lines. Values represent means +/- SE. Asterisks indicate significant differences between mock and dex-treated samples at the 95% confidence interval, as determined by Student's *t* test.

As shown in Figure 5A, expression of F-box mRNA was evident in dex-treated dex-βC1 plants, but not in mock-treated plants. Thus, transgenic expression of βC1 reactivated an endogenous, transcriptionally silenced locus. It should be noted that βC1 expression proved highly toxic, and dex-βC1 plants were mostly necrotic by 6 days post-treatment. Thus it was necessary to conduct these experiments with extracts obtained three days after a single dex treatment. It is probably for this reason that we were unable to observe expression of the additional silenced loci examined in our previous study (the transposons *Athila*, *AtSN1*, and *CACTA-like*). These earlier experiments involved multiple dex treatments spanning 9 to 17 days to drive expression of CaLCuV AC2/AL2 or BCTV C2/L2 transgenes [22]. We speculate that silencing at the *F-box* locus may be reversed more rapidly than at the transposons tested.

However, with extracts obtained three days after a single dex treatment, it was possible to observe the impact of transgenic βC1 expression on genome-wide CNG methylation using the single nucleotide extension assay described earlier. This analysis showed that with DNA from plants of two independent transgenic lines (βC1-L25 and βC1-L31), dex-induced expression of βC1 resulted in a ∼1.5-fold increase in the incorporation of labeled cytosine, indicating a global reduction of cytosine methylation at CNG sites (Figure 5B). The differences between dex- and mock-treated plants were significant at the 95% confidence interval. Together, these results further demonstrate that βC1 is able to suppress methylation and reverse TGS.

### βC1 interacts *in vivo* with SAHH

We reasoned that βC1 might inhibit methylation and TGS by interacting with components of the cellular methylation pathway or methyl cycle enzymes. Thus, we initiated a candidate screen using a yeast two-hybrid system that relies on *HIS3* and *ADE2* reporter genes to indicate interaction between proteins fused to the GAL4 DNA binding domain (BD, bait) or the GAL4 activation domain (AD, prey).

**Figure 6:**
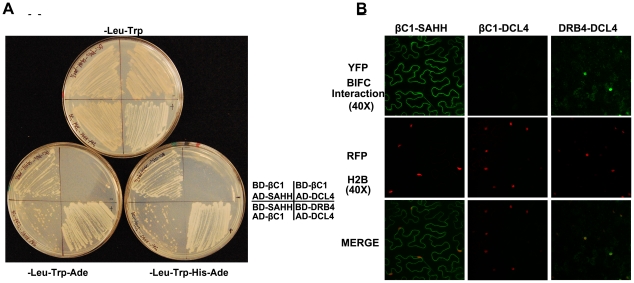
βC1 protein interacts with SAHH. (A) Yeast two-hybrid interaction. Bait proteins were expressed as GAL4 DNA binding domain fusions, and prey proteins as GAL4 activation domain fusions, in yeast PJ649A cells. Growth on media lacking leucine (-Leu) and tryptophan (-Trp) indicates maintenance of the bait and prey plasmids. Interaction is indicated by growth on plates also lacking adenine (-Ade), or histidine and adenine (-His-Ade). (B) BiFC interaction. Constructs expressing βC1 and SAHH fused to the N- or C-terminal portions of YFP were delivered to *N. benthamiana* leaf cells by agroinfiltration. Cells were photographed 48 hours post-infiltration at 40 X magnification using a confocal laser scanning microscope. RFP-histone 2B (RFP-H2B) was used as a marker for the nucleus. The co-expressed proteins are indicated above the photographs, which are representative of results with all possible combinations of fusion proteins. In both yeast two-hybrid and BiFC experiments, DRB4 was co-expressed with DCL4 as a positive control, and βC1 was expressed with DCL4 as a negative control.

Several *Arabidopsis* proteins have been tested to date, including the dsRNA binding proteins DRB2, DRB3, DRB4, and DRB5, the methyl cycle enzymes ADK and SAHH, and the Dicer-like proteins DCL2, DCL3, and DCL4. In our hands, full-length DCL3 expression proved lethal to yeast cells, so in this case a truncated protein consisting of the RNaseIII and dsRNA binding domains was used. DCL4 was co-expressed with DRB4 as a positive control [41]. Interestingly, βC1 protein was found to interact only with SAHH, an essential methyl cycle enzyme required for methylation-mediated TGS [29]. Yeast growth indicative of interaction was observed regardless of which protein was bait or prey (Figure 6A). To verify the specificity of βC1-SAHH interaction, SAHH was then tested and failed to interact with the DCL proteins, the DRB proteins, and ADK (data not shown).

SAHH is a cytoplasmic protein, and we previously showed that while the 14 kDa βC1 is enriched in the nucleus when expressed as a βC1-GFP fusion, a fraction remains in the cytoplasm [14]. Thus there is opportunity for these proteins to interact *in vivo*, and bimolecular fluorescence complementation (BiFC) using yellow fluorescent protein (YFP) was carried out to determine if and where it occurs. Constructs were designed to express βC1 or *Arabidopsis* SAHH fused at their N- or C- termini with the N- or C- terminal portions of YFP (YN and YC). Similar constructs containing the DCL4 and DRB4 proteins were used as controls. Expression constructs were introduced into *N. benthamiana* leaf cells by agroinfiltration, and those with opposite YFP fusions (i.e. YN + YC) were viewed under a confocal microscope 48 hours post-infiltration [27]. Histone 2B fused with red fluorescent protein (RFP-H2B) served as a marker for the nucleus. Test proteins were examined in all possible combinations.

No signal was detected in control experiments in which only one fusion protein was expressed, or when βC1 was co-expressed with DCL4. Co-expression of the DCL4 and DRB4 control proteins resulted in YFP fluorescence indicating that complex accumulation was mostly nuclear, although some signal was also apparent in the cytoplasm. When oppositely fused βC1 and SAHH were co-expressed, strong YFP fluorescence was observed in the cytoplasm (Figure 6B). We concluded that βC1 and SAHH specifically interact and form mainly cytoplasmic complexes *in vivo*.

### βC1 inhibits SAHH activity *in vitro*

**Figure 7:**
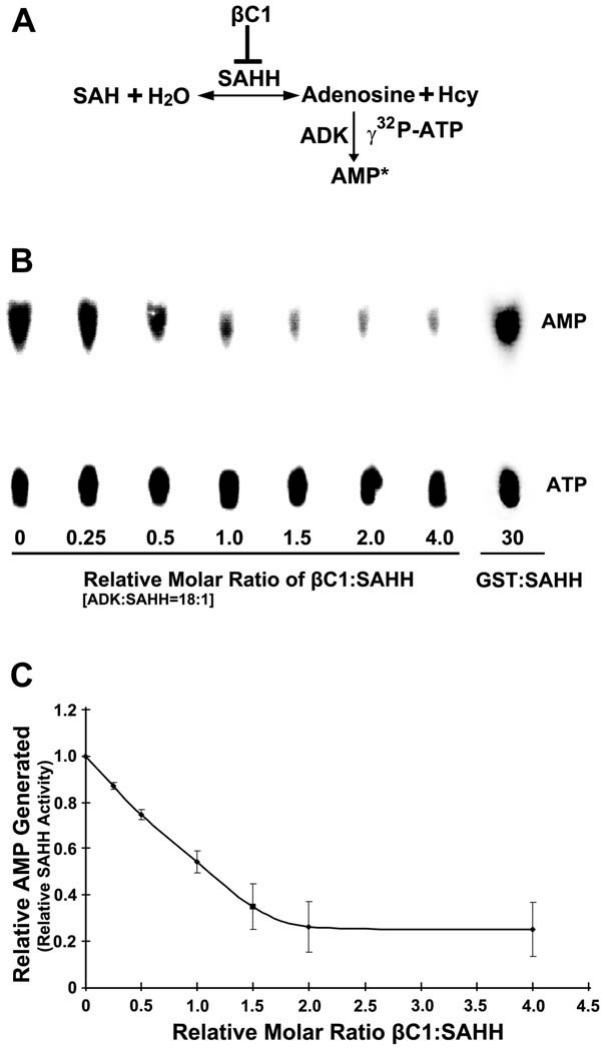
βC1 inhibits SAHH activity *in vitro*. (A) Diagram of the assay. In a reversible reaction, S-adenosyl homocysteine (SAH) is hydrolyzed by SAHH to adenosine and homocysteine (Hcy). Conversion of adenosine to labeled adenosine monophosphate (AMP*) by ADK in the presence of labeled γ^32^P-ATP drives the reaction in the direction of hydrolysis and allows indirect quantitation of the SAHH reaction. SAH hydrolysis promotes flux through the methyl cycle and SAM production, and this is inhibited by βC1 protein. (B) Autoradiograph of a representative chromatogram showing AMP generated by ADK in reactions containing varying molar ratios of βC1 to SAHH. The positions of labeled AMP product and ATP substrate are indicated. ADK activity (AMP/AMP+ATP) in each reaction was calculated after phosphorimager quantitation of radioactivity in individual spots. GST:SAHH (30∶1) served as a control. (C) Stoichiometry of inhibition. The graph illustrates relative ADK activity, an indirect measure of SAHH activity, with increasing βC1:SAHH molar ratio. Data were obtained from three independent experiments with two independent SAHH preparations. Values represent means +/− SE.

To assess the consequences of the βC1-SAHH interaction, we devised an indirect assay for SAHH activity. SAHH catalyzes the hydrolysis of S-adenosyl-homocysteine (SAH) to homocysteine (Hcy) and adenosine. However, the equilibrium for this reaction lies in the direction of SAH synthesis. To drive the reaction in the direction of hydrolysis, the direction that promotes flux through the methyl cycle, ADK was added to convert one of the hydrolytic products, adenosine, to adenosine monophosphate (AMP) in the presence of γ^32^P-ATP. The addition of ADK mimics the natural methyl cycle, and allowed us to indirectly quantify SAHH activity by measuring the amount of labeled AMP produced (Figure 7A). *Arabidopsis* SAHH and ADK2 were expressed as N-terminal, double hemagglutinin peptide, six histidine (HA_2_His_6_) fusions in *N. benthamiana* cells using a *Tobacco mosaic virus*-based TRBO vector [42], and proteins were purified from leaf extracts by nickel-NTA chromatography. While it was possible to express βC1 in the same manner, its toxicity limited yield. Thus a glutathione S-transferase fusion protein (GST-βC1) was expressed in *Escherichia coli* BL21 cells and purified by glutathione-agarose chromatography.

SAHH was pre-incubated for 20 minutes with varying amounts of βC1 (no βC1 to a four-fold molar excess βC1:SAHH) and a large molar excess of ADK (18∶1). βC1 does not interact with ADK in yeast cells (above), and control experiments confirmed that βC1 has no effect on ADK activity (data not shown). Protein mixtures were then added to solutions containing SAH, γ^32^P-ATP, and MgCl_2_ and reactions were allowed to proceed for 20 minutes, after which ADK activity was blocked by the addition of EDTA. Conversion of adenosine to AMP was monitored by thin layer chromatography (Figure 7B) [25]. Experiments with two independent preparations of SAHH showed that βC1 reduced enzyme activity by nearly 80% in this assay, with maximal inhibition at a molar ratio approaching 2∶1 (Figure 7C). In control reactions containing GST rather than GST-βC1, molar ratios as great as 30∶1 GST:SAHH did not diminish the amount of AMP produced. Thus, βC1 and SAHH interact directly *in vitro*, and SAHH activity is inhibited by this physical interaction. Moreover, inhibition of AMP formation with increasing βC1 is approximately linear in the dose-response range (R^2^ = 0.9965), indicating that inhibition of SAHH is stoichiometric.

To obtain evidence for the biological relevance of SAHH inhibition by βC1, we attempted to determine whether SAHH-deficient *Arabidopsis* plants showed enhanced susceptibility to TYLCCNV and BCTV *L2^-^*. Total loss of SAHH activity is lethal. However, the *hog1-1* alleles described by Rocha *et al*. are leaky and can be made homozygous. These mutants have reduced methyl cycle activity and as a result display severe methylation defects, developmental defects and a slow growth phenotype [29]. Unfortunately, the developmental abnormalities made it impossible to determine whether susceptibility to the viruses was altered (data not shown). However, in previous work, we found that other methyl cycle-deficient mutants, namely *adk1* and *adk2*, show greatly enhanced susceptibility to geminiviruses [20]. In addition, by using a *Tobacco rattle virus* (TRV) vector to promote gene silencing by RNA interference, we showed that knock-down of SAHH or ADK reversed TGS in *N. benthamiana* line 16-TGS [22].

### A silencing-defective mutant βC1 protein fails to interact with SAHH

In previous work, we analyzed a mutant βC1 protein (βC1^3A^) in which three lysine residues in the nuclear localization signal (^49^KKK^51^) were replaced by alanine. A betasatellite containing this mutation (TYLCCNB-3A) was trans-replicated by TYLCCNV but did not induce disease symptoms in *N. benthamiana* plants. Further, when transiently expressed, the βC1^3A^ protein failed to target the nucleus and was unable to suppress local GFP silencing (post-transcriptional gene silencing). Thus it was concluded that nuclear localization is required for silencing activity [14]. Since SAHH is a cytoplasmic enzyme, it was of interest to determine whether interaction with SAHH was affected by the alanine substitutions within the NLS, and whether TYLCCNB-3A or the βC1^3A^ mutant protein could suppress TGS.

**Figure 8:**
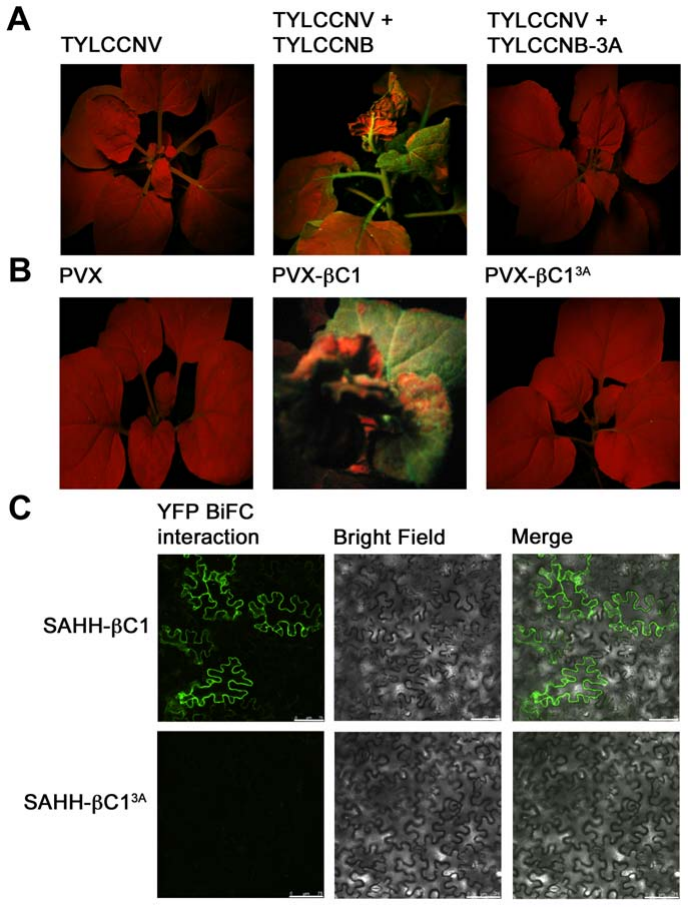
A mutation in βC1 blocks both TGS reversal and SAHH interaction. *N. benthamiana* plants containing a transcriptionally silenced GFP transgene (line 16-TGS) were inoculated as indicated and photographed under UV light 14 days post-inoculation. (A) Line 16-TGS plants were inoculated with TYLCCNV, or co-inoculated with TYLCCNV + TYLCCNB (expressing wild-type βC1) or TYLCCNB-3A, expressing βC1 in which lysine residues 49-51 are replaced by alanine (βC1^3A^). (B) Line 16-TGS plants were inoculated with the PVX vector, PVX expressing wild-type βC1 (PVX-βC1), or mutant βC1 (PVX-βC1^3A^). (C) BiFC interaction. Constructs expressing βC1, βC1^3A^, and SAHH fused to the N- or C-terminal portions of YFP were delivered to *N. benthamiana* leaf cells by agroinfiltration. Cells were photographed 48 hours post-infiltration using a confocal laser scanning microscope. The co-expressed proteins are indicated to the left of the photographs.

TYLCCNV was co-inoculated with either TYLCCNB or TYLCCNB-3A to *N. benthamiana* line 16-TGS plants as described earlier. As before, plants inoculated with TYLCCNV + TYLCCNB showed severe symptoms by 14 days post-inoculation and robust GFP expression, indicating reversal of TGS, was evident under UV light. By contrast, plants inoculated with TYLCCNV + TYLCCNB-3A did not display symptoms and showed no evidence of TGS reversal (Figure 8A). To confirm the absence of TGS suppression activity, βC1^3A^ was also expressed from a PVX vector. Again, while abundant yellow-green fluorescence was observed in 16-TGS plants inoculated with a vector expressing wild-type βC1, (PVX-βC1), none was visible in plants inoculated with PVX-βC1^3A^ (Figure 8B). Finally, BiFC analysis confirmed cytoplasmic interaction between wild-type βC1 and SAHH, and showed that βC1^3A^ was no longer capable of interacting with SAHH in *N. benthamiana* cells, despite the fact that it is confined to the cytoplasm [14] (Figure 8C). Taken together, these results suggest that the alanine substitutions within the NLS of βC1^3A^ also compromise its ability to interact with SAHH and suppress TGS, and provide additional evidence that cytoplasmic βC1-SAHH interaction is responsible for TGS reversal.

## Discussion

Our recent work has demonstrated that the small RNA-directed methylation pathway, which plays a key role in transposon control and the establishment of heterochromatin, also serves as a potent defense against geminiviruses. In particular, recovery from geminivirus infection, which is characterized by symptom remission, reduced virus replication, and hypermethylation of viral genomes, requires the methylation pathway [20]. We also observed that the geminivirus TYLCCNV systemically infects *N. benthamiana*, *Solanum lycopersicum*, and *Petunia hybrida* without inducing obvious disease, whereas symptoms are readily apparent following co-inoculation of these hosts with TYLCCNV and its associated betasatellite TYLCCNB [10]. This led us to hypothesize that plants might employ methylation as a defense against TYLCCNV, and that the βC1 protein encoded by TYLCCNB might suppress methylation and TGS. This study presents several lines of evidence in support of this hypothesis.

Using bisulfite sequencing, we found that cytosine residues in the TYLCCNV genome are methylated in infected *N. benthamiana* plants, and that methylation is enriched in regions that encompass or are adjacent to RNA polymerase II promoters. These include the IR, sequences within the AC1/AL1 coding region that also serve as promoters for transcripts that specify AC2/AL2 and AC3/AL3, and sequences within the AV2 and 5′-AV1/CP coding region that span the AV1/CP promoter [30], [31], [32], [33], [34]. Whether methylation *in vivo* reduces TYLCCNV transcription has yet to be directly examined, however, given the repressive effect of *in vitro* methylation of geminivirus DNA on replication and transcription in protoplasts, TGS is a likely outcome [24], [43]. How promoter sequences are preferentially targeted is unclear, especially since some are embedded within coding regions. However, the AV2 and AV1/CP region shown here to be a methylation target has also been identified as a hotspot for siRNA production in TYLCCNV infected *N. benthamiana* and *S. lycopersicum* plants [35]. It will be interesting to study the connection between TYLCCNV-derived siRNA and viral genome methylation, and to identify the sequences and/or proteins that direct the methylation system to promoters.

The ability of TYLCCNB, and specifically its βC1 protein, to inhibit methylation was demonstrated in several ways. First, bisulfite sequencing of the TYLCCNV genome following co-infection with TYLCCNB showed that methylation of the helper virus was substantially reduced at all locations, with the striking exception of the AV2/AV1 sequence noted above. We also showed that BCTV and BCTV *L2^-^* mutants can support low level replication of TYLCCNB, and that the betasatellite reduced methylation in all sequence contexts within the BCTV IR. Using a methylation-sensitive extension assay, we observed that TYLCCNB also caused global reductions in host genome cytosine methylation. That similar reductions in host genome methylation were observed following expression of βC1 protein from a PVX vector, and after transgenic expression of βC1 in *Arabidopsis*, provided direct evidence that methylation inhibition by TYLCCNB was due to this sole betasatellite-encoded protein. Similar experiments also revealed that TYLCCNB or βC1 can reverse TGS. Thus, in co-infections with TYLCCNV or BCTV *L2^-^*, TYLCCNB was observed to reverse TGS of a GFP transgene in *N. benthamiana* line 16-TGS. Suppression of GFP-directed TGS was also apparent following expression of βC1 from a PVX vector, and transgenic expression of βC1 reactivated an endogenous *Arabidopsis F-box* locus known to be transcriptionally silenced.

Because the TYLCCNV genome is methylated to a greater extent in the absence of TYLCCNB, and because TYLCCNV by itself is unable to reverse TGS, we speculated and showed that its AC2/AL2 protein is deficient for methylation inhibition and TGS suppression activities. Here our studies with BCTV are instructive. Like TYLCCNV, BCTV *L2^-^* mutants are unable to prevent viral genome hypermethylation or reverse TGS. As a result, wild-type *Arabidopsis* and *N. benthamiana* plants typically recover from BCTV *L2^-^* infection [20], [22]. Our demonstration that TYLCCNB complements BCTV *L2^-^* mutants by preventing host recovery and viral genome hypermethylation, and by reversing TGS, models the relationship between TYLCCNB and its cognate helper virus. We propose that a primary role of βC1 protein is to limit methylation-mediated TGS directed against the helper virus genome, and that this is a key factor stabilizing TYLCCNV-TYLCCNB association. The assignment of suppression functions to TYLCCNB βC1 might allow TYLCCNV AC2/AL2 to more efficiently perform other tasks. This implies that betasatellites have co-evolved with their helper viruses, and in a previous study we obtained direct evidence for this by showing that clustering of begomovirus sequences corresponds to clustering of betasatellite sequences. Further, the linear correlation (R^2^ = 0.8772) between pairwise nucleotide sequence identities of begomovirus and betasatellite DNAs clearly demonstrated that betasatellites have co-evolved with their cognate helper viruses [44].

To date, betasatellites have been found to be trans-replicated only by begomoviruses. Our observation that BCTV and BCTV *L2^-^* can support replication of a betasatellite, although at much reduced levels compared to the cognate helper virus TYLCCNV, is the first demonstration of betasatellite replication by a curtovirus. It is interesting that both BCTV and BCTV *L2^-^* appeared to replicate TYLCCNB to similar levels, despite the much greater accumulation of wild-type BCTV DNA (Fig. 2 B and 2C). We speculate that this may be because the wild-type virus, which does not require βC1 complementation to suppress silencing, can easily spread into new tissues without the satellite.

If the role of βC1 protein is to inhibit methylation, why was robust methylation of TYLCCNV DNA not observed when the virus was inoculated without TYLCCNB? We believe there are two reasons for this, one biological and one technical. First, the rolling circle and recombination-directed replication modes employed by geminiviruses may allow nascent viral genomes to escape significant methylation. Second, the PCR primers used to amplify bisulfite treated viral DNA were biased toward converted (unmethylated) templates. Thus, our data likely underestimates the amount of viral genome methylation in infected cells (Figure 1). In any case, the presence of TYLCCNB clearly reduced TYLCCNV methylation, and previous work has shown that even modest reductions in viral genome methylation result in dramatic increases in viral disease symptoms [20].

In addition to showing that methylation inhibition is a key βC1 function, we also provide evidence that this is accomplished by βC1-SAHH interaction. *SAHH/HOG1* (*HOMOLOGY-DEPENDENT GENE SILENCING 1*) was previously identified in a genetic screen as an activity required for methylation-mediated TGS [29]. The results of yeast two-hybrid and BiFC studies described here indicate that βC1 and SAHH specifically interact and form predominantly cytoplasmic complexes in *N. benthamiana* cells. Subsequent *in vitro* SAHH activity assays with purified proteins confirmed that the interaction is direct, and showed that physical interaction results in a stoichiometric and substantial reduction in SAHH activity. Why incomplete inhibition was observed *in vitro* is not clear, but it is likely that inhibition is limited by βC1:SAHH complex dissociation. The situation *in vivo* is additionally clouded by our ignorance of relative βC1:SAHH levels in infected cells. But in any case, as the absence of SAHH activity is lethal [29], partial inhibition might be a compromise that maximizes virus yield. Evidence for the relevance of βC1-SAHH interaction comes from our previous observations that methyl cycle-deficient mutants show enhanced susceptibility to geminiviruses [20], and that SAHH knock-down by virus induced gene silencing causes TGS reversal in *N. benthamiana* line 16-TGS [22]. Experiments in this report further demonstrate that a mutation in βC1^3A^ that compromises SAHH interaction also abolishes TGS suppression activity.

**Figure 9:**
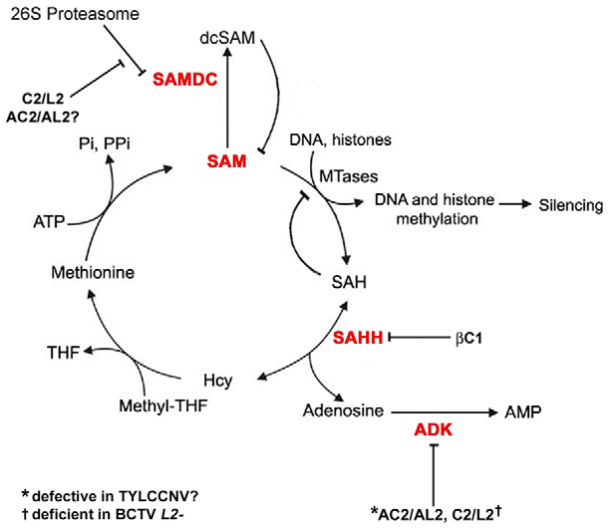
Model for methyl cycle inactivation by geminivirus and betasatellite proteins. S-adenosyl methionine (SAM) is the methyl donor for most transmethylation reactions. The product, S-adenosyl homocysteine (SAH), inhibits transmethylation by competing with SAM for methyltransferases (MTases). SAH is converted to homocysteine (Hcy) and adenosine by S-adenosyl homocysteine hydrolase (SAHH). Phosphorylation of adenosine by adenosine kinase (ADK) is critical because the SAHH-catalyzed reaction is reversible and the equilibrium lies in the direction of SAH synthesis. By removing adenosine, ADK promotes flux through the cycle and SAM production, and minimizes competitive inhibition of methyltransferase reactions by SAH. Thus, ADK inactivation by geminivirus AC2/AL2 and C2/L2 proteins globally interferes with methylation. Data presented in this report suggests that TYLCCNV AC2/AL2 may be deficient for ADK inhibition. The C2/L2 protein has also been shown to stabilize SAM decarboxylase (SAMDC), which causes decarboxylated SAM (dcSAM) levels to rise. dcSAM is a competitive inhibitor of SAM. Whether AC2/AL2 proteins also promote increases in dcSAM has yet to be tested. Data in this report indicates that the betasatellite encoded βC1 protein directly antagonizes the methyl cycle by inhibiting SAHH. Note that this diagram lists only DNA and histones as methyltransferase substrates, although any type of transmethylation reaction may require SAM as a cofactor. THF: tetrahydrofolate, PPi: pyrophosphate; Pi: inorganic phosphate.

Geminivirus employ multiple strategies to inhibit methylation and TGS. The AC2/AL2 proteins of CaLCuV, TGMV, and other begomoviruses suppress silencing by inactivating ADK, and by activating host genes that negatively regulate silencing pathways [22], [26], [45]. The related C2/L2 proteins of curtoviruses such as BCTV and BSCTV similarly inactivate ADK, and also inhibit proteasome-mediated degradation of SAMDC [23]. It is striking that these proteins, and the geminivirus-associated βC1, target the methyl cycle by multiple mechanisms (Figure 9). Thus it appears that reducing the availability or activity of SAM is an effective approach to interfering with host methylation-dependent defenses that may be widespread among virus families. Methylation interference via methyl cycle inhibition is not expected to be virus-specific. Accordingly, host genome-wide reduction of CNG methylation, and ectopic expression of endogenous loci normally silenced by methylation, was observed following geminivirus/betasatellite infection, and transgenic expression of viral and betasatellite suppressor proteins [22] (this report).

In summary, we have identified βC1 protein encoded by a geminivirus associated betasatellite as a new TGS suppressor, reinforcing the importance of methylation-mediated TGS as an epigenetic defense against geminiviruses. We propose that βC1 suppression of methylation and TGS drives geminivirus-betasatellite association, and further show that suppression is accomplished by βC1-SAHH interaction, which dramatically inhibits the activity of this essential methyl cycle enzyme.

## Materials and Methods

### Plant materials and virus inoculation

*N. benthamiana* plants, and *N. benthamiana* line 16-TGS, which contains a transcriptionally silenced green fluorescent protein (GFP) transgene [22], were grown in an insect-free chamber or in limited access growth rooms at 25°C with a 16∶8-hour (light/dark) photoperiod. Plants at the 4-6 leaf stage were agroinoculated with TYLCCNV (isolate Y10), TYLCCNB or TYLCCNB mutant (pBin-Y10βC1/3A), BCTV (strain Logan), BCTV *L2-1* or *L2-2* mutants, or PVX vectors as previously described [10], [14], [36], [46].

### PVX vector construction

To generate recombinant PVX, the *βC1* gene or NLS-mutated *βC1* were amplified by PCR using the full-length infectious clone pBinPLUS-2β or pBin-Y10βC1/3A as template. PCR products were first cloned into a pGEM-T Easy vector (Promega, Madison, WI) and subsequently into the PVX-containing pgR106 vector between the ClaI and SalI sites. The pgR106 vector was the kind gift of David Baulcombe [38]. The resulting plasmid PVX-βC1 and PVX-βC1^3A^ were mobilized into the *Agrobacterium tumefaciens* strain GV3101 by electroporation.

### Recovery and TGS reversal assays

Recovery assays were performed as described [20], [22]. Briefly, *N. benthamiana* wild-type or line 16-TGS plants were agroinoculated with BCTV or BCTV *L2^-^* mutant virus alone or in combination with TYLCCNB. After the primary harvest, plants were allowed to continue growing under the same conditions and symptom development was observed in new secondary shoots after an additional 2 to 3 weeks. GFP expression was evaluated using a 100 W hand held long-wave UV lamp (UV products, Upland, CA) and photographed with a Nikon 5000 digital camera (Tokyo, Japan) equipped with UV and yellow filters.

### DNA isolation, bisulfite sequencing, and DNA blot hybridization

Genomic DNA was extracted from plant leaf samples using the DNeasy Plant Mini kit (Qiagen, Valencia, CA). To improve the efficiency of bisulfite treatment, DNA (1 µg) was digested with a restriction enzyme that cuts outside the region of interest to decrease the size of DNA, followed by overnight treatment with proteinase K. Bisulfite modification was carried out using the EZ DNA Methylation Gold™ kit (Zymo Research, Irvine, CA) in a PCR machine. Bisulfite-modified DNA was purified using a Zymo-Spin™ IC column and dissolved in 10 µl of Elution Buffer according to the manufacturer's instructions. PCR amplification was then carried out using ZymoTaq. PCR products were cloned into a pGEM-T easy vector (Promega), and individual clones were sequenced with an automated model 3730 DNA Sequencer (Perkin-Elmer, Foster City, CA). Primers were designed against converted templates and are listed in Table S1. To ensure bisulfite modification was complete, plasmids containing TYLCCNV DNA were added to mock inoculated plant DNA extracts and used for bisulfite treatment. Bisulfite sequencing of the BCTV IR was performed similarly and has been previously described [20], [47]. DNA blot hybridization to assess viral DNA accumulation was carried out essentially as described [20].

### Methylation sensitive extension assay

Global DNA methylation status was evaluated using a cytosine extension assay [37]. Briefly, 1 µg of genomic DNA was digested overnight with a 10-fold excess of MspI (C ^↓^CGG; New England Biolabs, Beverly, MA), leaving an overhang at non-methylated sites. A second DNA aliquot from mock inoculated samples was incubated without restriction enzyme and served as a background control. Single-nucleotide-extension reactions with α^32^P-dCTP and Taq DNA polymerase were then performed as previously described [22].

### Transgenic *Arabidopsis* studies

Transgenic lines expressing βC1 were obtained by transforming the Columbia ecotype of *A. thaliana* with construct pTA00-βC1, containing the βC1 coding sequence under the control of a dexamethasone (dex) inducible promoter. The *βC1* gene was first amplified and cloned into pGEM-T Easy vector (Promega) to derive pGEM-βC1, and then cloned into pTA7001, containing the dex-inducible promoter, as a SpeI-XhoI fragment [48]. Transformation of *Arabidopsis* plants was performed using the floral dip method [49]. Transgenic lines were selected on Murashige and Skoog medium (Invitrogen, Carlsbad, CA) containing B vitamins and 30 µg/ml hygromycin. Hygromycin-resistant, homozygous T2 plants were identified and used for experiments. Dex-induction treatments were performed essentially as described [22]. *Arabidopsis* plants were sprayed 3 to 5 weeks post-germination with 20 µM dex (Invitrogen) or mock-treated, and leaves were harvested and prepared to analyze transgene expression and its effects.

### RNA extraction and analysis

Total RNA was extracted with Trizol reagent (Invitrogen) from systemically infected plant leaves, or leaves of transgenic dex-βC1 plants, following the manufacturers' recommendations. For RNA blot analysis of GFP mRNA, 5–10 µg of total RNA was separated on 1.2% formaldehyde-agarose gels, transferred to Hybond N^+^ membranes (GE Healthcare, Bucks, UK) and UV cross-linked. Hybridization and subsequent washing was performed as described [14], and washed membranes were exposed using a Typhoon 9200 imager (Amersham Pharmacia, Piscataway, NJ) or a Phosphorimager (Bio-Rad Molecular Imager FX, Hercules, CA) for hybridization signal detection. For semi-quantitative RT-PCR (sqRT-PCR) analysis, 1 µg of total RNA was reverse transcribed into cDNA using the Superscript III RT-PCR system (Invitrogen). A fragment of actin mRNA was amplified as a control. PCR was carried out for 25 cycles, and amplified products were separated on 1.5% agarose gels and visualized under UV light. The primers used for sqRT-PCR are listed in Table S1.

### βC1-SAHH interaction assays

Yeast two-hybrid studies were carried out essentially as described [50], using the MATCHMAKER system (Clonetech, Mountain View, CA). Bait proteins were expressed as GAL4 DNA binding domain fusions in pAS2, and prey proteins as GAL4 activation domain fusions in pACT2, in yeast PJ649A cells. Bimolecular fluorescence complementation (BiFC) was carried using previously described vectors and methods [27]. PCR primers were used to amplify genes with the introduction of a PacI site at the 5′ end and an AscI site at the 3′ end. The PCR products were subsequently digested with PacI and AscI and ligated into similarly digested yeast vectors pAS2 and pACT2, and BiFC vectors p2YN, p2YC, pYC and pYN.

### SAHH activity assays

*Arabidopsis* SAHH and ADK2 proteins were expressed in *N. benthamiana* cells using a TRBO vector [42], and purified from leaf extracts by nickel-NTA chromatography. Cloning of SAHH and ADK2 cDNAs has been described, as has expression of ADK2 in TRBO [22], [42]. βC1 was PCR amplified, inserted into pGEX (GE Healthcare Life Sciences), expressed as a glutathione S-transferase fusion protein (GST-βC1) in *E. coli* BL21 cells, and purified by glutathione-agarose chromatography. Primers used for amplification of βC1 and SAHH prior to insertion in expression vectors are given (Table S1).

Indirect assays of SAHH activity were carried out according to the scheme presented in Figure 7A, using AMP production by ADK as a measure of SAHH-catalyzed SAH hydrolysis that yields adenosine and homocysteine. Mixtures containing, in a total volume of 15 µl, 10 ng SAHH, 180 ng ADK and various amounts of βC1 were pre-incubated at 30°C for 20 minutes. Mixtures were then added to reactions containing (final concentrations) 50 mM Tris-HCl, pH 7.6, 5 µM SAH (Sigma, St. Louis, MO), 5 µCi γ^32^P-ATP (3000 Ci/mmol), and 10 mM MgCl_2_. Reactions were incubated at 30°C for 20 minutes, when ADK activity was terminated by addition of 1 µl of 1M EDTA. AMP production was analyzed by thin layer chromatography on polyethyleneimine cellulose plates developed with 1 M acetic acid [25]. After chromatography, radioactive signals on plates were quantitated using a phosphorimager (Bio-Rad Molecular Imager FX).

## Supporting Information

Figure S1 Bisulfite sequencing of clones from TYLCCNV genome fragments MeA1 to MeA6. *N. benthamiana* plants were inoculated with TYLCCNV alone or co-inoculated with TYLCCNV+TYLCCNB. Methylation of the six TYLCCNV fragments was assessed by bisulfite sequencing. Twenty clones were sequenced for each fragment. The circles represent cytosines in each fragment and are color coded according to sequence context (red CG, green CHH, blue CNG). Filled circles indicate methylation, and each line represents the sequence of an individual clone. PCR primers used to amplify viral DNA fragments are listed in Table S1.
(PDF)

Figure S2 TYLCCNV AC2 expression cannot reverse TGS of a GFP transgene. (A) 16-TGS plants were inoculated with the PVX vector, PVX expressing AC2 from TYLCCNV (PVX-AC2), or PVX expressing βC1 (PVX-βC1), and were photographed under UV light 14 days post-inoculation. Results shown are representative of at least three independent experiments with 4 to 8 plants per treatment. (B) Gel blot analysis of RNA from leaves of 16-TGS plants inoculated as indicated. The ^32^P-labeled probe was specific for GFP mRNA. The 18S rRNA loading controls were visualized by ethidium bromide staining.
(PDF)

Table S1 Sequences of primers used in this study.
(PDF)
