# Supplementary material for: Suppression of Methylation-Mediated Transcriptional Gene Silencing by βC1-SAHH Protein Interaction during Geminivirus-Betasatellite Infection
Source: PLoS Pathog. 2011 Oct 20;7(10):e1002329. doi: 10.1371/journal.ppat.1002329 (PMC3197609; doi:10.1371/journal.ppat.1002329)
Supplement: Figure S1 — Bisulfite sequencing of clones from TYLCCNV genome fragments MeA1 to MeA6. N. benthamiana plants were inoculated with TYLCCNV alone or co-inoculated with TYLCCNV+TYLCCNB. Methylation of the six TYLCCNV fragments was assessed by bisulfite sequencing. Twenty clones were sequenced for each fragment. The circles represent cytosines in each fragment and are color coded according to sequence context (red CG, green CHH, blue CNG). Filled circles indicate methylation, and each line represents the sequence of an individual clone. PCR primers used to amplify viral DNA fragments are listed in Table S1. (PDF) [file ppat.1002329.s001.pdf]

Figure S1

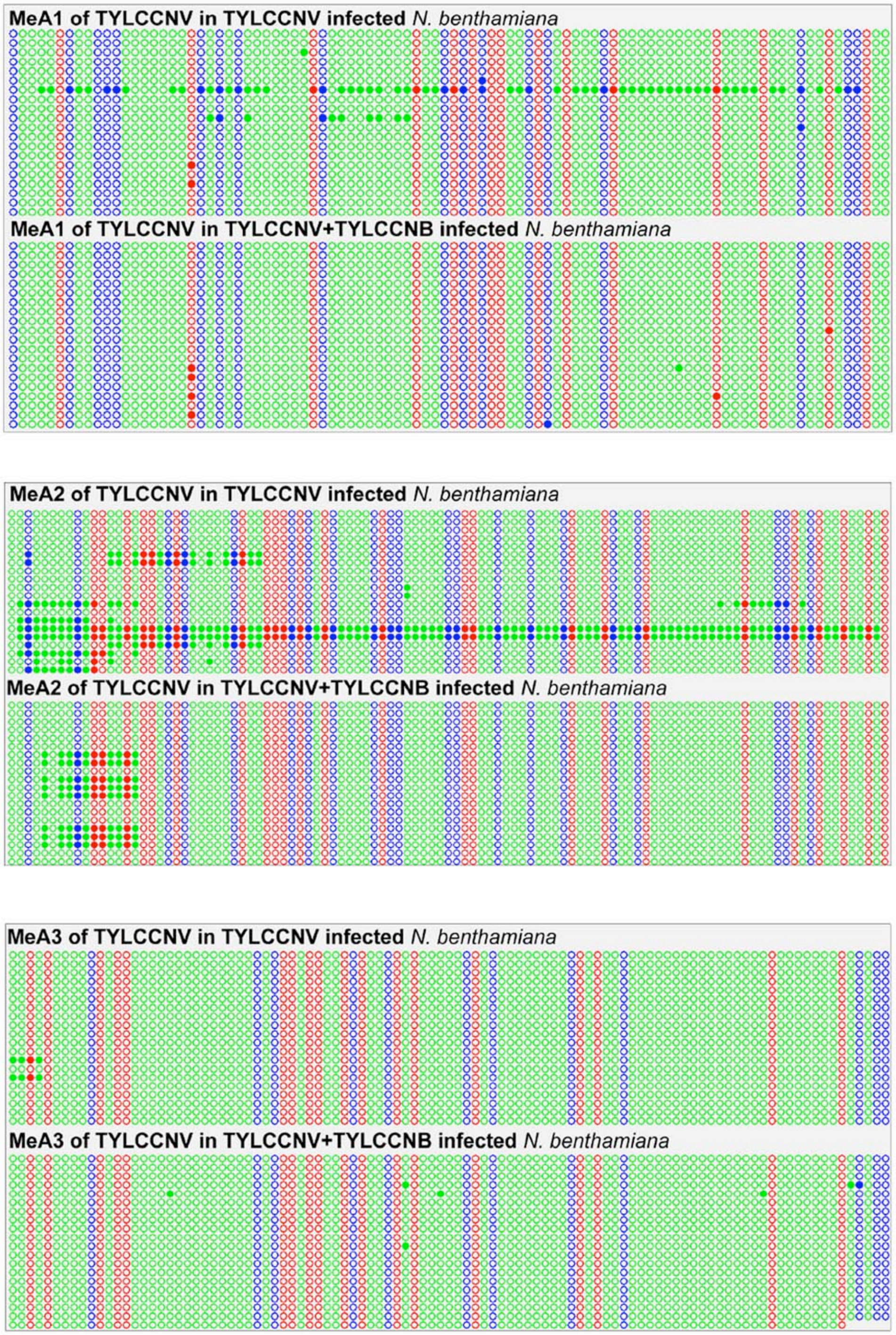
MeA1 of TYLCCNV in TYLCCNV infected N. benthamiana
MeA1 of TYLCCNV in TYLCCNV+TYLCCNB infected N. benthamiana
MeA2 of TYLCCNV in TYLCCNV infected N. benthamiana
MeA2 of TYLCCNV in TYLCCNV+TYLCCNB infected N. benthamiana
MeA3 of TYLCCNV in TYLCCNV infected N. benthamiana
MeA3 of TYLCCNV in TYLCCNV+TYLCCNB infected N. benthamiana

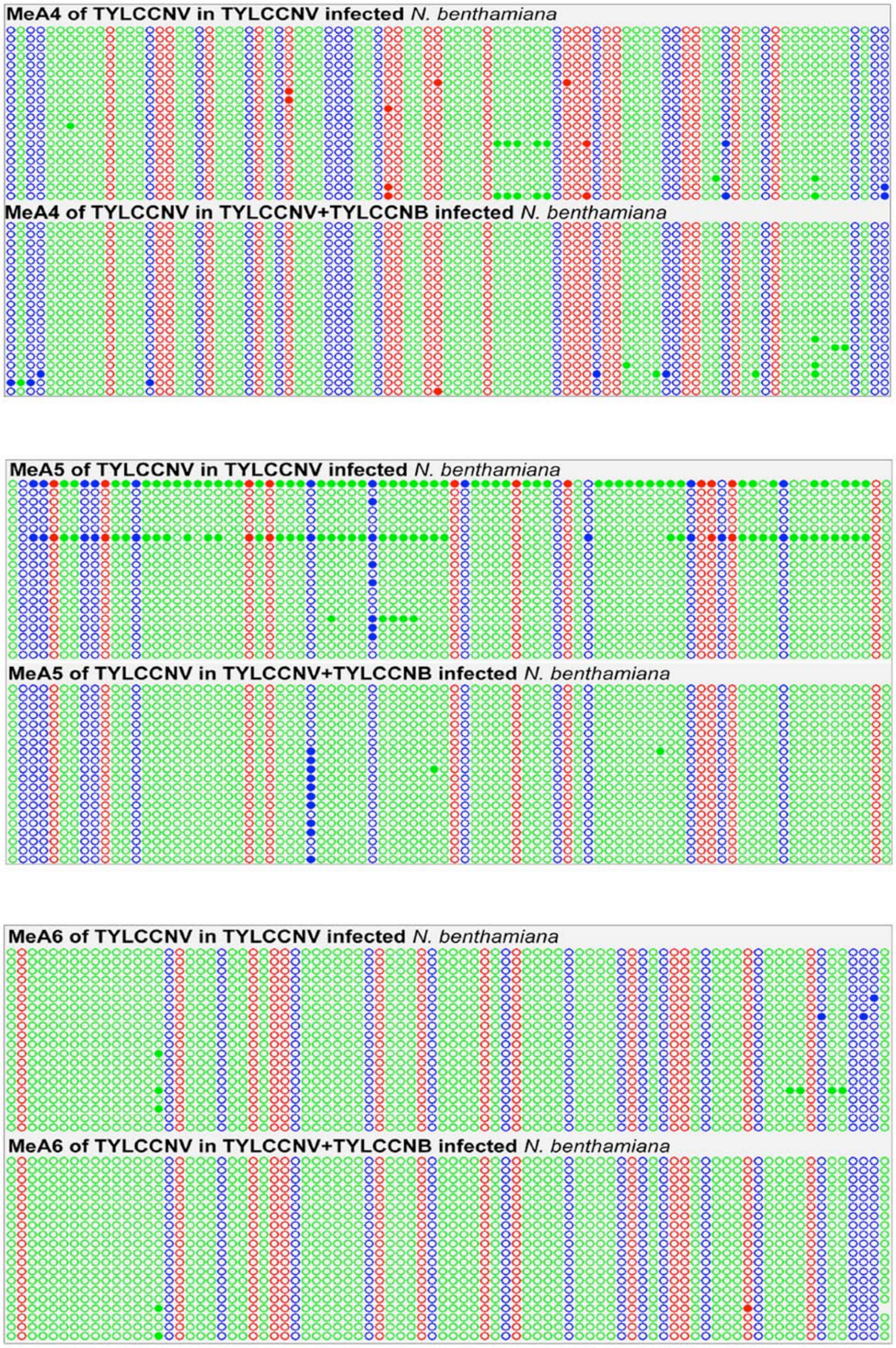

Figure S1. Bisulfite sequencing of clones from TYLCCNV genome fragments MeA1 to MeA6. *N. benthamiana* plants were inoculated with TYLCCNV alone or co-inoculated with TYLCCNV+TYLCCNB. Methylation of the six TYLCCNV fragments was assessed by bisulfite sequencing. Twenty clones were sequenced for each fragment. The circles represent cytosines in each fragment and are color coded according to sequence context (red CG, green CHH, blue CNG). Filled circles indicate methylation, and each line represents the sequence of an individual clone. PCR primers used to amplify viral DNA fragments are listed in Table S1.
