# Supplementary material for: Suppression of Methylation-Mediated Transcriptional Gene Silencing by βC1-SAHH Protein Interaction during Geminivirus-Betasatellite Infection
Source: PLoS Pathog. 2011 Oct 20;7(10):e1002329. doi: 10.1371/journal.ppat.1002329 (PMC3197609; doi:10.1371/journal.ppat.1002329)
Supplement: Figure S2 — TYLCCNV AC2 expression cannot reverse TGS of a GFP transgene. (A) 16-TGS plants were inoculated with the PVX vector, PVX expressing AC2 from TYLCCNV (PVX-AC2), or PVX expressing βC1 (PVX-βC1), and were photographed under UV light 14 days post-inoculation. Results shown are representative of at least three independent experiments with 4 to 8 plants per treatment. (B) Gel blot analysis of RNA from leaves of 16-TGS plants inoculated as indicated. The 32P-labeled probe was specific for GFP mRNA. The 18S rRNA loading controls were visualized by ethidium bromide staining. (PDF) [file ppat.1002329.s002.pdf]

Figure S2

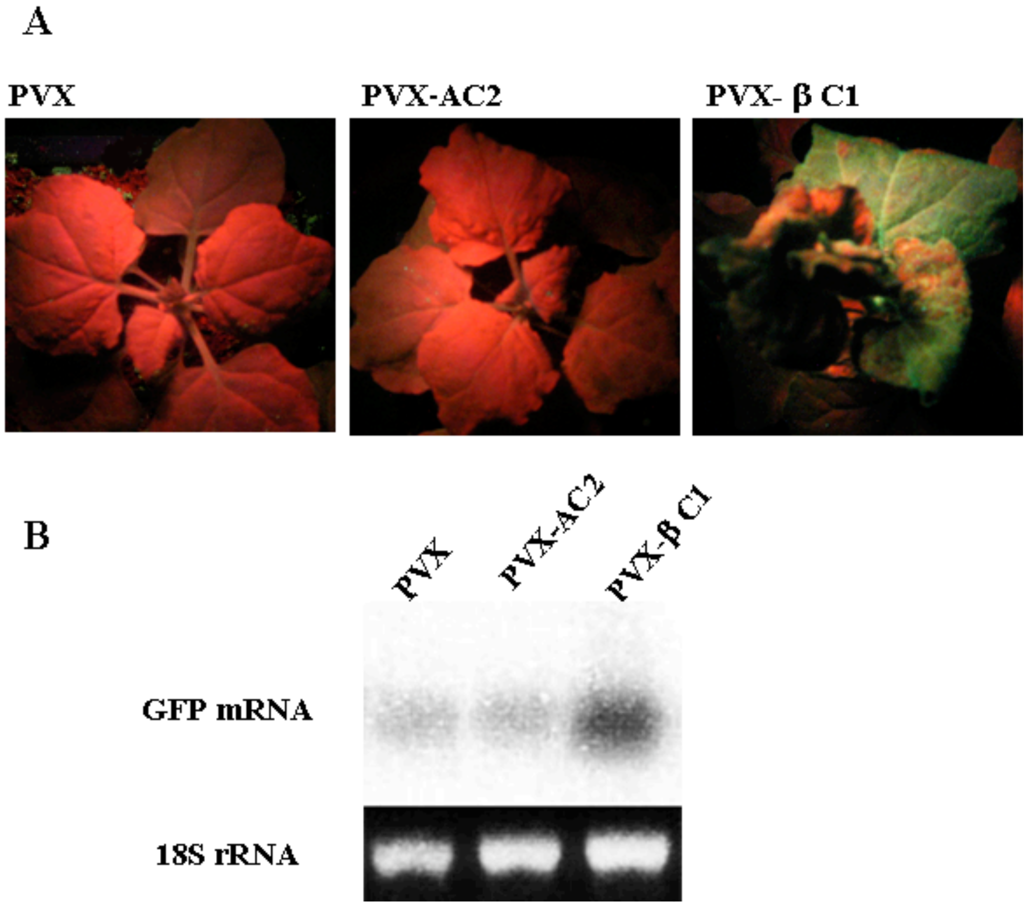

Figure S2. TYLCCNV AC2 expression cannot reverse TGS of a GFP transgene. (A) 16-TGS plants were inoculated with the PVX vector, PVX expressing AC2 from TYLCCNV (PVX-AC2), or PVX expressing βC1 (PVX-βC1), and were photographed under UV light 14 days post-inoculation. Results shown are representative of at least three independent experiments with 4 to 8 plants per treatment. (B) Gel blot analysis of RNA from leaves of 16-TGS plants inoculated as indicated. The ^{32}P-labeled probe was specific for GFP mRNA. The 18S rRNA loading controls were visualized by ethidium bromide staining.
