# Supplementary material for: Suppression of Methylation-Mediated Transcriptional Gene Silencing by βC1-SAHH Protein Interaction during Geminivirus-Betasatellite Infection
Source: PLoS Pathog. 2011 Oct 20;7(10):e1002329. doi: 10.1371/journal.ppat.1002329 (PMC3197609; doi:10.1371/journal.ppat.1002329)
Supplement: Table S1 — Sequences of primers used in this study. (PDF) [file ppat.1002329.s003.pdf]

TABLE S1. Sequences of primers used in this study

Primer	Primer sequence (5′-3′)	Length
MeA1/F	ATTGGATGAGGATATGTAGGTGAG	24
MeA1/R	TACTTAATTACTAACATACACCTAAAACCA	30
MeA2/F	GTATATGGTTTTAGGTGTATGTTAG	25
MeA2/R	TCTCATCCATCCATATCTTCCCA	23
MeA3/F	GTGTTGGTAAGAGGTTTTGTGTTA	24
MeA3/R	CCTACAAAATCTAAACTCATTTACATCCT	29
MeA4/F	AGGTTTGAGGATGTAAATGAGT	22
MeA4/R	ACCCACAATCTTCCTCTACAATCC	24
MeA5/F	TGGAATTGGGTTTTGGATTG	20
MeA5/R	CTCCTCCAATTAACAAATATATTTCTCCATT	31
MeA6/F	TGGTTAAAAGAAGAATAAGAAAATGGAG	28
MeA6/R	CCCTAAAAACCCCAATTACTAAAT	24
Y10βC1-ClaI/F	ATCGATATGACTATCAAATACAAC	24
Y10βC1-SalI/R	GTCGACTCATACATCTGAATTTG	23
Y10βC1-XhoI/F	CTCGAGACTATCAAATACAAC	21
Y10βC1-SpeI/R	ACTAGTTCATACATCTGAATTTG	23
F-box RT/F	AAAATCAGGTTCTGGAAAGG	20
F-box RT/R	TTCCCAGAGATCAGCATTGG	20
Actin RT/F	AGTATTGTTGGTCGTCCTAG	20
Actin RT/R	AAGAACTGCTCTTGGCTGTC	20
Y10βC1-PacI/F	TTAATTAACATGACTATCAAATACAAC	27
Y10βC1-AscI/R	GGCGCGCCCTACATCTGAATTTG	23
SAHH-PacI/F	TTAATTAACATGGCGTTGCTCGTCGAGAAG	30
SAHH-AscI/R	GGCGCGCCCTCAGTACCTGTAGTGAGGAGGC	31
TRBO-SAHH/F	GCGGGATCCTTAATTAAGGCCATGGCGTTGCTCGTCGAG	39
TRBO-SAHH/R	GCGCTGCAGGCGCGCCACTAGTCAGTACCTGTAGTGAGG	39
GST-βC1/F	GCGGATCCTTAATTAAGGACATGTATCATCCACAACAAA	39
GST-βC1/R	GCGTCTAGAGGCGCGCCCTACATCTGAATTTGTAAATAC	39
